# 5-Episinuleptolide Decreases the Expression of the Extracellular Matrix in Early Biofilm Formation of Multi-Drug Resistant *Acinetobacter baumannii*

**DOI:** 10.3390/md14080143

**Published:** 2016-07-29

**Authors:** Sung-Pin Tseng, Wei-Chun Hung, Chiung-Yao Huang, Yin-Shiou Lin, Min-Yu Chan, Po-Liang Lu, Lin Lin, Jyh-Horng Sheu

**Affiliations:** 1 Department of Medical Laboratory Science and Biotechnology, College of Health Sciences, Kaohsiung Medical University, Kaohsiung 807, Taiwan; cookiecandy760428@hotmail.com (Y.-S.L.); min116min@yahoo.com.tw (M.-Y.C.); 2 Department of Marine Biotechnology and Resources, National Sun Yat-sen University, Kaohsiung 804, Taiwan; huangcy@mail.nsysu.edu.tw; 3 Department of Microbiology and Immunology, Kaohsiung Medical University, Kaohsiung 807, Taiwan; wchung@kmu.edu.tw; 4 Department of Laboratory Medicine, Kaohsiung Medical University Hospital, Kaohsiung 807, Taiwan; d830166@cc.kmu.edu.tw; 5 College of Medicine, Kaohsiung Medical University, Kaohsiung 807, Taiwan; 6 Department of Internal Medicine, Kaohsiung Medical University Hospital, Kaohsiung 807, Taiwan; 7 Department of Culinary Art, I-Shou University, Kaohsiung 840, Taiwan; chang1025@msn.com; 8 Department of Medical Research, China Medical University Hospital, China Medical University, Taichung 404, Taiwan; 9 Asia Pacific Ocean Research Center, National Sun Yat-sen University, Kaohsiung 804, Taiwan; 10 Frontier Center for Ocean Science and Technology, National Sun Yat-sen University, Kaohsiung 804, Taiwan

**Keywords:** 5-Episinuleptolide, biofilm, multi-drug resistant *A. baumannii*

## Abstract

Nosocomial infections and increasing multi-drug resistance caused by *Acinetobacter baumannii* have been recognized as emerging problems worldwide. Moreover, *A. baumannii* is able to colonize various abiotic materials and medical devices, making it difficult to eradicate and leading to ventilator-associated pneumonia, and bacteremia. Development of novel molecules that inhibit bacterial biofilm formation may be an alternative prophylactic option for the treatment of biofilm-associated *A. baumannii* infections. Marine environments, which are unlike their terrestrial counterparts, harbor an abundant biodiversity of marine organisms that produce novel bioactive natural products with pharmaceutical potential. In this study, we identified 5-episinuleptolide, which was isolated from *Sinularia leptoclados*, as an inhibitor of biofilm formation in ATCC 19606 and three multi-drug resistant *A. baumannii* strains. In addition, the anti-biofilm activities of 5-episinuleptolide were observed for Gram-negative bacteria but not for Gram-positive bacteria, indicating that the inhibition mechanism of 5-episinuleptolide is effective against only Gram-negative bacteria. The mechanism of biofilm inhibition was demonstrated to correlate to decreased gene expression from the *pgaABCD* locus, which encodes the extracellular polysaccharide poly-β-(1,6)-*N*-acetylglucosamine (PNAG). Scanning electron microscopy (SEM) indicated that extracellular matrix of the biofilm was dramatically decreased by treatment with 5-episinuleptolide. Our study showed potentially synergistic activity of combination therapy with 5-episinuleptolide and levofloxacin against biofilm formation and biofilm cells. These data indicate that inhibition of biofilm formation via 5-episinuleptolide may represent another prophylactic option for solving the persistent problem of biofilm-associated *A. baumannii* infections.

## 1. Introduction

*Acinetobacter baumannii* is a Gram-negative, non-fermenting bacillus that is primarily found in healthcare and hospital environments. Recently, nosocomial infections and multi-drug resistance have been identified as emerging global problems [1,2]. Multi-drug resistant and pan-resistant *A. baumannii* strains have expanded globally and have now been found throughout the world, and only a few antibiotics can effectively treat these bacterial infections [3]. The major concern with *A. baumannii* is its ability to colonize various abiotic materials and medical devices, as it can cause ventilator-associated pneumonia, bacteremia, and wound infections [4,5]. Biofilm formation aids bacterial survival in strict nutrient-limiting environments and decreases their susceptibility to antibiotics [1,6]. In *A. baumannii*, the *pgaABCD* locus encodes proteins required for the production of poly-β-(1,6)-*N*-acetylglucosamine (PNAG), an extracellular polysaccharide that is important for biofilm formation [7]. PNAG has been shown to be a major component of *Staphylococcus aureus*, *S. epidermidis* and *Escherichia coli* biofilms [8,9,10]. Furthermore, PNAG is an important virulence factor that protects bacteria from the host innate immune system [11]. The *bla*_PER-1_ gene is an extended-spectrum β-lactamase-encoding gene that is associated with cell adhesiveness and biofilm formation in *A. baumannii* [12]. Biofilm-associated protein (Bap) also plays an important role in colonization and biofilm formation [13,14,15]. The Bap protein is characterized as a large protein containing multiple tandem repeats that is able to anchor onto a cellular surface. Recent studies have found that the Bap protein is involved in initial adherence, biofilm maturation and maintenance in *A. baumannii* [16,17].

Biofilm formation serves as a barrier that significantly decreases the penetration of drugs into the biofilm, and bacteria within the biofilm are thus exposed to sub-lethal concentrations of antibiotics [18]. Sub-lethal concentrations of antibiotics increase mutation rates and the likelihood of plasmids and transposon transfer, thus selecting for resistant bacterial cells [19,20]. Thus, the development of anti-biofilm compounds could be an effective alternative strategy for treating biofilm infections. PGG (1,2,3,4,6-penta-*O*-galloyl-β-d-glucopyranose) is an active antioxidant ingredient found in geraniums, which are commonly used in Chinese medicine to treat inflammation [21,22]. PGG decreased biofilm formation by inhibiting initial attachment and the synthesis of PNAG [23]. Pinkston et al. developed a monoclonal antibody that targets a major component of *Enterococcus faecalis* pili (EbpC) and found that it diminished biofilm formation [24]. Weng et al. reported a quorum sensing inhibitor (F5) derived from *Pseudomonas* spp. JM2 that interferes with the *las* system (quorum sensing system) and significantly inhibits biofilm formation in *P. aeruginosa* [25]. However, only one study reported that a small organic molecule (virstatin) inhibits pili biosynthesis to prevent biofilm formation by *A. baumannii* [26].

**Figure 1:**
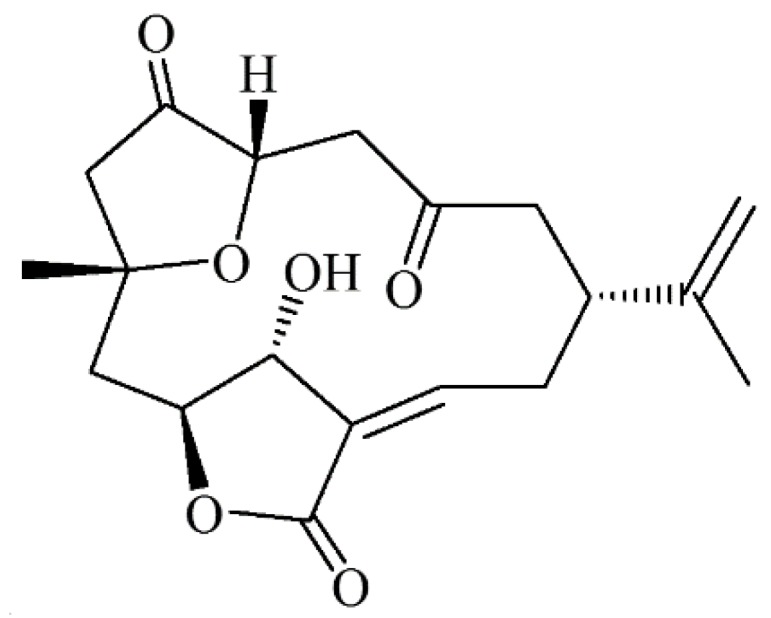
Chemical structure of 5-episinuleptolide.

Marine natural products have demonstrated pharmaceutical potential, particularly for the development of anticancer [27], antiviral [28] and antibacterial drugs [29]. The soft corals of the *Sinularia* genus are well known for producing bioactive and structurally versatile natural compounds [30]. The norcembranoids sinuleptolide and 5-episinuleptolide (Figure 1) have been isolated from the Formosan soft corals *Sinularia scabra* [31], *S. parva* [32], and *S. leptoclados* [32]. Later, they were again isolated from the coral *S. lochmodes* and the absolute molecular structures of both isomers were established [33]. Both norcembranoids were shown to inhibit LPS-induced TNF-α and nitric oxide production in murine macrophage RAW 246.7 cells [34], and they also inhibited the growth of several cancer cell lines [31]. Previous studies have demonstrated that bacteria can survive in hostile conditions, such as nutrient limitation and the presence of antimicrobial compounds via biofilm formation [4,5,6]. Indeed, the efficacy of anti-biofilm drugs has not yet been established in clinical biofilm-associated infections. In this study, 5-episinuleptolide was tested for its ability to inhibit biofilm formation, and the mechanisms of biofilm inhibition were investigated in multi-drug resistant *A. baumannii* strains. Furthermore, we determined that 5-episinuleptolide, isolated from *S. leptoclados*, could diminish biofilm formation by multi-drug resistant *A. baumannii*.

## 2. Results

### 2.1. Efficacy of 5-Episinuleptolide against A. baumannii Biofilm Formation Rather Than Eradication of Biofilm

**Figure 2:**
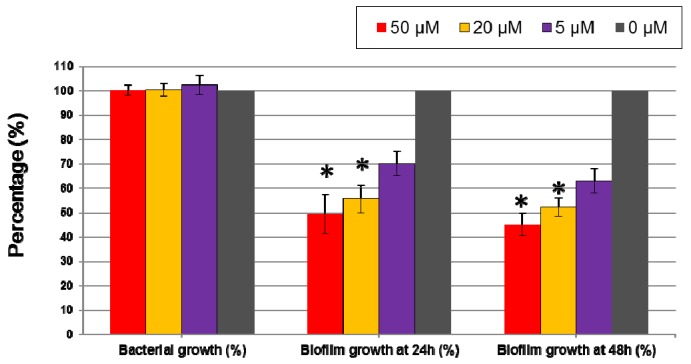
Effect of 5-episinuleptolide on biofilm formation of *Acinetobacter baumannii* ATCC 19606. Biofilm formation was determined by a microtiter plate assay with TSB medium supplemented with 1% glucose. An untreated control set as 100% in bacterial growth and biofilm growth. Experiments were performed in triplicate.

To test the activity of 5-episinuleptolide against *A. baumannii* biofilm formation and eradication of biofilm, the effect of bacterial growth was first tested with *A. baumannii* ATCC 19606. At 50 μM, 20 μM, and 5 μM, 5-episinuleptolide did not inhibit bacterial growth at 24 h, a result that was confirmed by plate count methods (at 50 μM, 9.1 ± 0.7 × 10^8^ CFU/mL; at 20 μM, 8.6 ± 0.5 × 10^8^ CFU/mL; at 5 μM, 8.8 ± 1.2 × 10^8^ CFU/mL and at 0 μM, 9.0 ± 1.1 × 10^8^ CFU/mL). The activity of anti-biofilm growth increased with increasing concentrations of 5-episinuleptolide, which showed significant anti-biofilm activity at 50 μM (49.38% at 24 h; 45.21% at 48 h) and 20 μM (55.58% at 24 h; 52.3% at 48 h) (*p* < 0.05) (Figure 2). The similar anti-biofilm activity at 24 h and 48 h indicated that 5-episinuleptolide could persist for 48 h. The eradication ability of 5-episinuleptolide was tested with *A. baumannii* ATCC 19606, and it was found that no significant differences among different concentrations of 5-episinuleptolide (data not shown). These results indicate that 5-episinuleptolide inhibits biofilm growth rather than eradicating biofilm.

### 2.2. Antimicrobial Susceptibility Testing of Multi-Drug Resistant A. baumannii Isolates

The results of antimicrobial susceptibility testing, organized into nine categories, are presented in Table 1. *Pseudomonas aeruginosa* ATCC 27853 and *E. coli* ATCC 25922 were used as quality control strains for the antibiotics. Among three clinical isolates, *A. baumannii* 29115 and 68704 were resistant to six antibiotics but were susceptible to colistin (Table 1). *A. baumannii* D4 was resistant to eight antibiotics and was susceptible to only colistin. Infections with multi-drug resistant *A. baumannii* isolates are difficult to treat, as many of the currently used antibiotics are not effective against these isolates. If these isolates were to form biofilms, further reduced antibiotic susceptibility would represent a serious treatment problem.

### 2.3. Inhibition of Biofilm Formation by 5-Episinuleptolide in Multi-Drug Resistant A. baumannii Clinical Isolates and Reference Strains

To test the inhibitory activity of 5-episinuleptolide against biofilm formation by multi-drug resistant *A. baumannii* clinical isolates, *A. baumannii* 29115, 68704 and D4 were employed. The ratios of inhibition for these isolates were similar to that for *A. baumannii* ATCC 19606 (Table 2). Biofilm growth was 59.9% (*A. baumannii* 29115), 45.83% (*A. baumannii* 68704), and 43.9% (*A. baumannii* D4) at 50 μM, and 61.07% (*A. baumannii* 29115), 87.13% (*A. baumannii* 68704), and 60.23% (*A. baumannii* D4) at 20 μM, respectively.

To investigate whether the inhibition of biofilm formation differed between Gram-negative and Gram-positive bacteria, four reference strains, including Gram-negative bacteria (*E. coli* ATCC 25922 and *P. aeruginosa* ATCC 33592) and Gram-positive bacteria (*S. aureus* ATCC 29213 and *S. epidermidis* RP62A) were tested. Anti-biofilm activities were observed with Gram-negative bacteria but not with Gram-positive bacteria (Table 2). These results indicated that the anti-biofilm activity of 5-episinuleptolide is active against Gram-negative bacteria but not against Gram-positive bacteria.

### 2.4. Investigation of the Biofilm Inhibitory Mechanism of 5-Episinuleptolide

**Figure 3:**
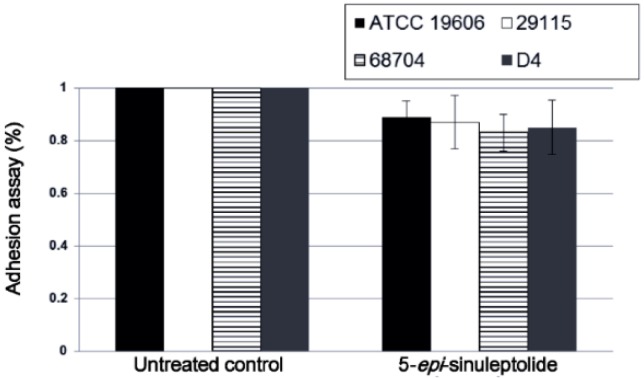
Effect of 5-episinuleptolide on bacterial adhesion. Bacterial strains were cultured in TSB_gluc1%_ broth with or without 5-episinuleptolide in 96-well microtiter plates at 37 °C for 4 h. Experiments were performed in triplicate.

Because Bap, PNAG, and quorum sensing are known to be important for biofilm formation [35,36], these parameters were analyzed using an adhesion assay, SEM and quantitative RNA expression analysis. There were no significant differences in adherence between the untreated control and the 5-episinuleptolide treated cells (Figure 3).

**Figure 4:**
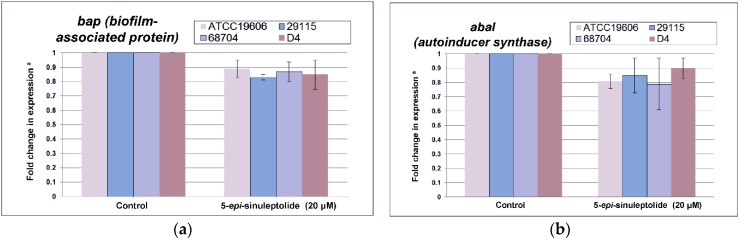

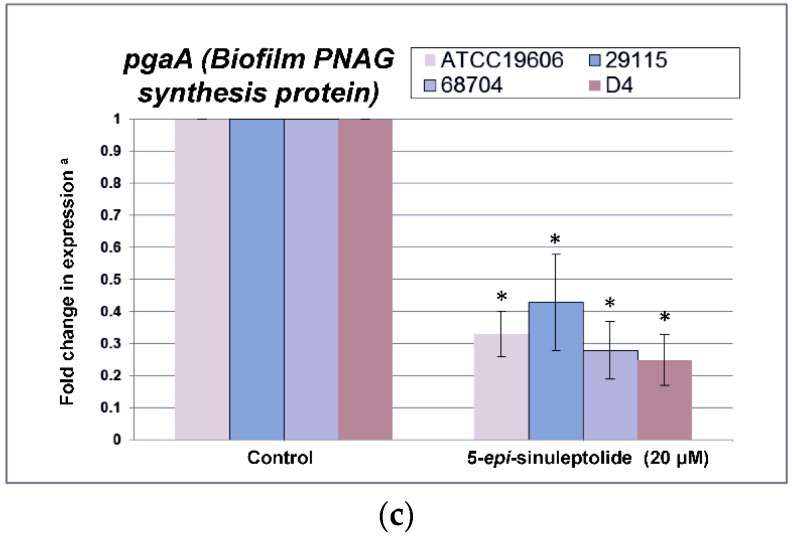
Quantitative RNA expression in *A. baumannii* strains treating with 5-episinuleptolide at 0 (control) and 20 μM concentrations. (**a**) fold change in *bap* expression; (**b**) fold change in *abaI* expression; and (**c**) fold change in *pgaA* expression. Experiments were performed in triplicate. * p < 0.05 using the Mann-Whitney *U* test.

**Figure 5:**
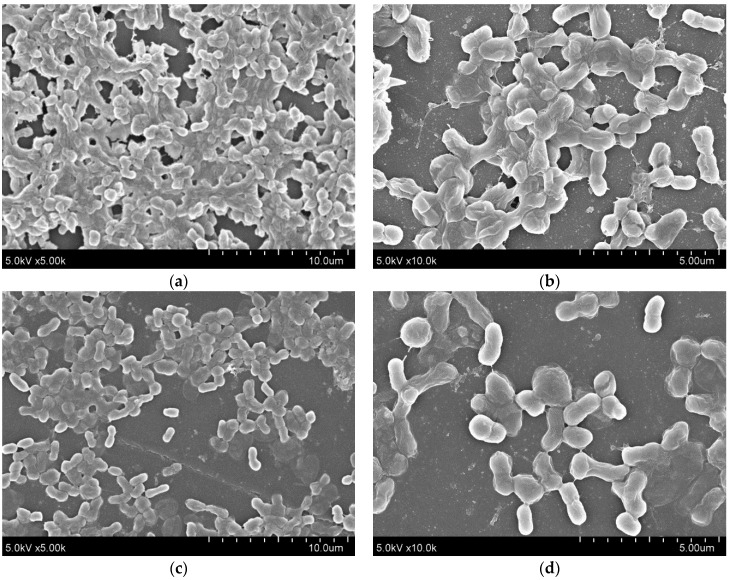
SEM images for biofilms of *A. baumannii* ATCC 19606 on polystyrene discs treating with 5-episinuleptolide at 0 and 20 μM concentrations. (**a**) untreated control at 5000× magnification; (**b**) untreated control at 10,000× magnification; (**c**) treating with 5-episinuleptolide (20 μM) at 5000× magnification; and (**d**) treating with 5-episinuleptolide (20 μM) at 10,000× magnification.

Furthermore, quantitative RNA expression analysis of *bap* showed that *bap* expression was not altered by 5-episinuleptolide treatment (Figure 4a). These data indicate that Bap is not the target of 5-episinuleptolide. The effect of the drug on the autoinducer synthase (AbaI) for quorum sensing was also analyzed by quantitative RNA expression. However, the expression of *abaI* was not altered by 5-episinuleptolide treatment, indicating that AbaI is not the target of 5-episinuleptolide (Figure 4b). SEM examination indicated that ATCC 19606 cells were completely embedded in extracellular polymeric substances in the untreated control (Figure 5a,b), while the amount of biofilm extracellular matrix was dramatically decreased by 5-episinuleptolide treatment at 20 μM (Figure 5 c,d). Quantitative RNA analysis showed that treatment with 5-episinuleptolide significantly decreased the expression of *pgaA* compared to the untreated control in all of the isolates (*p* < 0.05) (Figure 4c). These results indicated that inhibition of PNAG synthesis by 5-episinuleptolide may decrease the expression of the extracellular matrix.

### 2.5. Anti-Biofilm Efficacy of a Combination of 5-Episinuleptolide with Levofloxacin

Levofloxacin, which is one of the outstanding 3rd-generation of quinolone antibiotics treating a number of bacterial infections, was selected to test the efficacy of combination therapy. To investigate combination therapy with 5-episinuleptolide and levofloxacin against biofilm formation, four *A. baumannii* strains were employed. The inhibitory ratios of 5-episinuleptolide in combination with 0.5× MIC (minimum inhibitory concentration) levofloxacin were significantly reduced when treated with 50 and 20 μM 5-episinuleptolide (* *p* < 0.05) (Table 3). Biofilm growth was 25.62% (*A. baumannii* ATCC 19606), 33.46% (*A. baumannii* 29115), 27.47% (*A. baumannii* 68704), and 26.35% (*A. baumannii* D4) at 50 μM, and 31.83% (*A. baumannii* ATCC 19606), 40.58% (*A. baumannii* 29115), 47.46% (*A. baumannii* 68704), and 35.19% (*A. baumannii* D4) at 20 μM, respectively.

**Figure 6:**
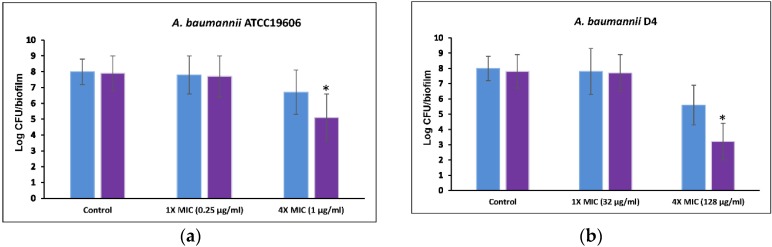
Efficacy of 5-episinuleptolide in combination with levofloxacin against biofilm cells. Untreated control (blue bars) and treating with 5-episinuleptolide (20 μM) (purple bars) were exposed to levofloxacin in various concentration. (**a**) *A. baumannii* ATCC 19606; and (**b**) *A. baumannii* D4. Experiments were performed in triplicate. * *p* < 0.05 using Mann-Whitney *U* test.

To test combination therapy with 5-episinuleptolide and levofloxacin against biofilm cells, two bacterial strains (ATCC 19606 and D4) were employed. No significant differences were observed between the untreated controls and ATCC 19606 and D4 cells treated with 20 μM 5-episinuleptolide (Figure 6a,b). When treated with 1× MIC levofloxacin, the biofilm cells of these strains also showed similar numbers between untreated controls and treatment with 20 μM 5-episinuleptolide. In the presence of 4× MIC levofloxacin, biofilm cells were significantly reduced when treated with 20 μM 5-episinuleptolide (*p* < 0.05). These results indicated a potentially synergistic activity of 5-episinuleptolide and levofloxacin against biofilm formation and biofilm cells.

## 3. Discussion

Antibiotic discovery is important for the treatment of infectious diseases. In the past century, different antibiotics have been discovered and successfully deployed against serious infections. However, the increasing prevalence of antibiotic-resistant bacteria has become a global health problem, i.e., bacteria are difficult to treat. Traditional antibiotic strategies that focus on killing bacteria or preventing bacterial growth have led to antibiotic resistance; however, inhibition of bacterial virulence and signaling pathways may represent alternative approaches for the treatment of bacterial infections [25,37,38].

Marine environments are unlike terrestrial environments, and the abundant biodiversity of marine organisms produce novel and bioactive natural products. In the past decade, more than 3000 newly identified marine-derived compounds have been reported having novel therapeutic uses [27]. Peach et al. found that auromomycin inhibits biofilm formation by *Vibrio cholerae* with an IC_50_ of 60.1 μM [39]. Halogenated furanones produced by the marine red alga *Delisea pulchra* have been reported to inhibit the quorum sensing systems of *Serratia liquefaciens*, *P. aeruginosa*, and *Bacillus subtilis* [40,41,42]. This is the first study that reports the anti-biofilm activity of 5-episinuleptolide, a secondary metabolite isolated from *Sinularia leptoclados*, against a reference strain and multi-drug resistant isolates of *A. baumannii* (Table 2). 5-Episinuleptolide at 20 μM concentration, which is negligibly harmful to nontransformed human skin fibroblast Hs68 cells (>95% cell survival) [43], could effectively inhibit biofilm formation. However, 5-episinuleptolide is an inhibitor of biofilm growth rather than a biofilm eradicator. These results suggest that applications of 5-episinuleptolide should be focused on biofilm prevention. Table 2 shows that anti-biofilm activities were observed for Gram-negative bacteria but not for Gram-positive bacteria. Most detailed analysis demonstrated that biofilm inhibition by 5-episinuleptolide involves decreased gene expression of the *pgaABCD* locus (Figure 4c). Although a quantitative RNA analysis showed the inhibition of PNAG synthesis by 5-episinuleptolide (Figure 4), quantitative PNAG protein was not detected in this study. Further experiments should be performed to confirm this finding.

Interestingly, 5-episinuleptolide did not inhibit biofilm formation in *S. aureus* ATCC 29213 and *S. epidermidis* RP62A (Table 2) even though they also produce PNAG. One reason for this difference may be due to PNAG being produced by a different gene locus in these species. A previous study revealed that *icaADBC* locus, which produced PNAG in *S. epidermidis*, is similar to *pgaABCD* locus in that they produce low protein [44]. The different protein sequences might explain different effects of 5-episinuleptolide on Gram-positive and Gram-negative bacteria. The absence of any significant difference in an adhesion assay indicated that initial step of biofilm formation (*CsuA*/*BABCDE*-mediated pili and Bap) was not targeted by 5-episinuleptolide (Figure 3 and Figure 4a). However, the autoinducer synthase (AbaI) of quorum sensing reportedly participates in biofilm maturation [45], the RNA expression of *abaI* was not altered by 5-episinuleptolide treatment, indicating that AbaI is not a target of 5-episinuleptolide (Figure 4b). Our previous study showed that tigecycline, imipenem-rifampicin and colistin-rifampicin would be effective for the prevention or reduction of biofilm formation in *A. baumannii* strains [46]. Our study revealed the potentially synergistic activity of 5-episinuleptolide and levofloxacin against biofilm formation (Table 3 and Figure 6a,b). A previous study revealed that 5-episinuleptolide caused the inhibition of TNF-α and nitric oxide production in macrophages from several cancer cell lines, indicating an effect of the immune system [25]. Although the immune system may be affected by treatment with 5-episinuleptolide, possibly making it unsuitable for direct use against infections, this new lead compound could have potential for further discovery of anti-biofilm agents.

The global expansion of multi-drug resistant and pan-resistant *A. baumannii* strains has resulted in such strains being found throughout the world [3]. Only a few effective antibiotics can treat these bacterial infections, supporting the importance of biofilm inhibition as an alternative therapeutic option for their treatment. Chabane et al. found that a small organic molecule (virstatin) inhibits pili biosynthesis to prevent biofilm formation in *A. baumannii* [26]. Our finding revealed that 5-episinuleptolide could decrease gene expression from the *pgaABCD* locus, which is an important component of biofilm formation. Thus, the synergistic anti-biofilm effects of virstatin (pili biosynthesis) and 5-episinuleptolide (PNAG biosynthesis) might find potential use for inhibition of *A. baumannii* biofilm formation.

## 4. Materials and Methods

### 4.1. Marine Natural Products

The compound of 5-episinuleptolide was isolated from *Sinularia leptoclados*, which was identified by Jyh-Horng Sheu, National Sun Yat-Sen University, Kaohsiung, Taiwan, as described previously [43]. 5-Episinuleptolide could induce apoptosis of human skin cancer cells [43]. The stock solution of 5-episinuleptolide was dissolved in dimethyl sulfoxide (DMSO) at a concentration of 100 mM. The stock solution was diluted to the desired final concentrations with growth medium immediately before use.

### 4.2. Bacterial Isolates

Two *A. baumannii* reference strains (ATCC 19606 and ATCC BAA747), *E. coli* ATCC 25922, *P. aeruginosa* ATCC33592, *S. aureus* ATCC 29213, *S. epidermidis* RP62A and three multi-drug resistance clinical isolates of *A. baumannii*, which were isolated from blood in Kaohsiung Medical University Chung-Ho Memorial Hospital in 2011, were analyzed in this study.

### 4.3. Ethics Statement

This study was approved by the Institutional Review Board (IRB) of Kaohsiung Medical University Chung-Ho Memorial Hospital, Kaohsiung, Taiwan (KMUHIRB-E(II)-20150159). The study subjects were bacterial isolates, and written consent was given by the patients was waived by the approving IRB.

### 4.4. Antimicrobial Susceptibility Testing

Antimicrobial susceptibility testing was performed by agar dilution according to the guidelines of the Clinical and Laboratory Standards Institute (CLSI) [35]. The MIC was defined as the lowest concentration of antibiotic that prevented bacterial growth after 20 to 24 h of incubation at 37 °C. The following antimicrobial agents were tested: ceftazidime, colistin, doxycycline, gentamicin, levofloxacin, meropenem, ticarcillin, ticarcillin-clavulanic acid, and trimethoprim-sulfamethoxazole.

### 4.5. Measurement of Bacterial Growth

Bacteria were cultured overnight at 37 °C in TSB broth and adjusted to a McFarland 1.0 with TSB_gluc1%_. Next, 1:50 dilutions were prepared with TSB_gluc1%_ and transferred to 96-well flat-bottomed microtiter plates (Corning Incorporated, Corning, NY, USA). After 24 h of incubation at 37 °C, the bacterial density was determined at A600 using a microtiter plate reader (Molecular Devices, Sunnyvale, CA, USA) and confirmed by plate count methods.

### 4.6. Adhesion and Biofilm Formation Assays

Biofilm formation was assayed using the microtiter plate assay [47]. Briefly, bacteria were cultured overnight at 37 °C in TSB broth and adjusted to a McFarland 1.0 with TSB_gluc1%_. Next, 1:50 dilutions were prepared with TSB_gluc1%_ and transferred to 96-well flat-bottomed microtiter plates (Corning Incorporated, Corning, NY, USA). Bacterial suspensions were incubated with 5-episinuleptolide at various concentrations (0, 5 μM, 20 μM, and 50 μM). After 4 h (adhesion assay) or 24 h and 48 h (biofilm formation assay) of incubation at 37 °C, the wells were washed twice with water and then dried. Biofilms were stained with 0.6% crystal violet for 1 min, washed twice with water to remove excess dye and then dried. The biofilm matrix was resuspended in 10% acetic acid, and the absorbance of the crystal violet was measured at OD_570_ nm. *S. epidermidis* RP62A was used as a positive control, and experiments were performed in triplicate.

### 4.7. Biofilm Eradication Assay

To test the eradication ability of 5-episinuleptolide, the biofilm formation assay was modified to create a biofilm eradication assay. Briefly, bacteria were cultured overnight at 37 °C in TSB broth and adjusted to a McFarland 1.0 with TSB_gluc1%_. Next, 1:50 dilutions were prepared with TSB_gluc1%_ and transferred to 96-well flat-bottomed microtiter plates (Corning Incorporated, Corning, NY, USA). After 24 h of biofilm growth at 37 °C, the wells were washed twice with water to remove non-adherent cells. Subsequently, the biofilm cells were treated with 5-episinuleptolide at various concentrations (0, 5 μM, 20 μM, and 50 μM) for 24 h at 37 °C. The remaining biofilms were evaluated with crystal violet as biofilm formation assay.

### 4.8. RNA Extraction and Synthesis of cDNA

Total RNA was extracted from bacterial biofilm cells (untreated controls and cells treated with 20 μM 5-episinuleptolide) using TRIzol reagent (Life Technologies, Carlsbad, CA, USA) according to the manufacturer’s recommendations. Contaminating DNA was removed by DNase I (Life Technologies, Carlsbad, CA, USA) digestion for 45 min at 37 °C, followed by phenol-chloroform extraction, isopropanol precipitation, and resuspension of total RNA in nuclease-free water. Reverse transcription was performed with 5 μg of RNA using random hexamers and M-MLV reverse transcriptase (Life Technologies, Carlsbad, CA, USA).

### 4.9. Quantitative Reverse Transcription-PCR (qRT-PCR) for Biofilm-Related Genes

PCR reactions were performed in 96-well plates in reaction buffer containing 1X FastStart Universal SYBR Green Master (Roche), 300 nM primers and 2 μL of cDNA. Reactions were conducted in an ABI7000 machine (Applied Biosystems, Carlsbad, CA, USA) following the manufacturer’s protocol. Forward and reverse primers were developed using Primer-BLAST (http://www.ncbi.nlm.nih.gov/tools/primer-blast/) and are listed in Table 4. Relative fold changes in the transcript levels of the indicated genes were normalized to the 16SrDNA gene (internal control) and calculated using the 2^−^*^△△CT^* method.

### 4.10. Analysis of Biofilm Formation by Scanning Electron Microscopy

Biofilms were grown on polystyrene discs and were treated with 5-episinuleptolide at 0 or 20 μM. After 24 h of incubation, the discs were washed three times with PBS to remove planktonic cells and were prepared for SEM examination as previously described [16]. Samples were analyzed with an SU8010 scanning electron microscope (Hitachi, Tokyo, Japan).

### 4.11. Anti-Biofilm Efficacy of 5-Episinuleptolide in Combination with Levofloxacin

A combination of 5-episinuleptolide and levofloxacin was tested using a previously described method [48]. Briefly, biofilms were grown for 24 h in TSB_gluc1%_ and treated with different concentrations of levofloxacin (1× MIC and 4× MIC were tested) in combination with 20 μM 5-episinuleptolide. Biofilms were grown on silicone discs (Q7-4735, Dow Corning, Midland, MI, USA) that were placed in the wells of a 24-well microtiter plate. After 24 h of treatment, all discs were rinsed and transferred to 10 mL of TSB_gluc1%_. Bacterial cells were removed from the discs by vortexing (30 s) and sonication (30 s in an ultrasound sonicator DC400H, Yu Xin Instrument Co. Ltd., Tainan, Taiwan). Subsequently, the number of bacterial cells was optimized to approximately 10^8^ cells/mL, which were determined by plate count methods. Experiments were performed in triplicate.

### 4.12. Statistical Analysis

The SPSS 15.0 program (SPSS, Inc., Chicago, IL, USA) was used for statistical analysis. A nonparametric test (the Mann-Whitney *U* test) was used to examine the difference in biofilm formation between the untreated control group and the 5-episinuleptolide treated group. A *p* < 0.05 threshold was considered significant for this analysis.

## 5. Conclusions

The natural compound 5-episinuleptolide, isolated from *S. leptoclados*, could diminish biofilm formation in ATCC 19606 and three multi-drug resistant *A. baumannii* strains. The mechanism of biofilm inhibition was attributed to decreased gene expression of the *pgaABCD* locus, whose products produce the extracellular polysaccharide poly-β-(1,6)-*N*-acetylglucosamine (PNAG). The use of 5-episinuleptolide may represent an alternative prophylactic option for solving the persistent problem of biofilm-associated *A. baumannii* infections.

## Figures and Tables

**Table 1:** Antimicrobial susceptibilities of *A. baumannii* isolates.

Antibiotics ^a^	*A. baumannii* Isolates				
	ATCC 19606	BAA747	29115	68704	D4
SXT	>8/152 (R)	<1/19 (S)	>8/152 (R)	>8/152 (R)	>8/152 (R)
DOX	<2 (S)	<2 (S)	64 (R)	64 (R)	64 (R)
TIM	128/2 (R)	16/2 (S)	32/2 (I)	32/2 (I)	256/2 (R)
CT	<1 (S)	<1 (S)	<1 (S)	<1 (S)	<1 (S)
CAZ	16 (I)	16 (I)	>128 (R)	>128 (R)	>128 (R)
MEM	<2 (S)	<2 (S)	32 (R)	64 (R)	>128 (R)
GM	32 (R)	<2 (S)	>128 (R)	>128 (R)	>128 (R)
LEV	<1 (S)	<1 (S)	16 (R)	32 (R)	32 (R)
TIC	128 (R)	16 (S)	32 (I)	32 (I)	128 (R)

^a^ SXT: Trimethoprim-sulfamethoxazole; DOX: Doxycycline; TIM: Ticarcillin-clavulanic acid; CT: Colistin; CAZ: Ceftazidime; MEM: Meropenem; GM: Gentamicin; LEV: Levofloxacin; TIC: Ticarcillin.

**Table 2:** Inhibition of biofilm formation by 5-episinuleptolide in multi-drugs resistant *A. baumannii* clinical isolates and four reference strains.

Strain	Bacterial Growth (%)			Biofilm Growth (%)		
	50 μM	20 μM	5 μM	50 μM	20 μM	5 μM
*A. baumannii*						
ATCC 19606	100.2 ± 2.1	100.4 ± 2.7	102.4 ± 4.1	49.38 ± 8.1 *	55.58 ± 5.6 *	70.02 ± 5.0
29115	96.2 ± 2.3	100.3 ± 4.4	103.1 ± 4.8	59.90 ± 6.1 *	61.07 ± 4.2 *	76.81 ± 8.7
68704	105.8 ± 7.2	98.4 ± 6.2	103.9 ± 4.9	45.83 ± 2.2 *	87.13 ± 5.4	96.33 ± 6.0
D4	102.4 ± 2.9	98.6 ± 2.3	97.7 ± 5.5	43.90 ± 3.8 *	60.23 ± 4.6 *	78.75 ± 6.7
*E. coli*						
ATCC 25922	99.5 ± 6.2	98.5 ± 3.7	100.4 ± 8.2	45.48 ± 7.7 *	50.18 ± 9.4 *	82.33 ± 7.1
*P. aeruginosa*						
ATCC 27853	100.5 ± 7.6	108.9 ± 6.1	102.8 ± 5.7	53.01 ± 3.8 *	77.82 ± 5.5	95.48 ± 9.2
*S. aureus*						
ATCC 29213	103.3 ± 9.3	99.7 ± 5.6	105.3 ± 9.2	90.25 ± 7.4	99.57 ± 6.8	103.82 ± 5.6
*S. epidermidis*						
RP62A	99.7 ± 9.5	99.4 ± 2.8	98.7 ± 7.4	88.98 ± 7.9	113.06 ± 10.1	116.19 ± 8.1

* *p* < 0.05 using Mann-Whitney *U* test.

**Table 3:** Inhibition of biofilm formation by 5-episinuleptolide in combination with 0.5× MIC (minimum inhibitory concentration) levofloxacin in four *A. baumannii* strains.

Strain	Biofilm Growth Without 0.5× MIC (%)			Biofilm Growth With 0.5× MIC (%)		
	50 μM	20 μM	5 μM	50 μM	20 μM	5 μM
*A. baumannii*						
ATCC 19606	49.38 ± 8.1 *	55.58 ± 5.6 *	70.02 ± 5.0	25.62 ± 7.3 *	31.83 ± 4.4 *	67.92 ± 8.4
29115	59.90 ± 6.1 *	61.07 ± 4.2 *	76.81 ± 8.7	33.46 ± 7.7 *	40.58 ± 8.7 *	69.15 ± 5.7
68704	45.83 ± 2.2 *	87.13 ± 5.4	96.33 ± 6.0	27.47 ± 6.8 *	47.46 ± 3.6 *	79.57 ± 8.6
D4	43.90 ± 3.8 *	60.2 3± 4.6 *	78.75 ± 6.7	26.35 ± 8.4 *	35.19 ± 6.3 *	71.73 ± 7.7

**Table 4:** Primers used in this study.

Target	Primer Name	Sequence (5′ to 3′)	Sizes (bp)	Reference
16SrDNA	16S-F	TCCTCCAGTTTGTCACTGGC	116	This study
	16S-R	GTCAGCTCGTGTCGTGAGAT		
*bap*	BAP-F	CCTTGGTAACCACAGAGGGA	114	This study
	BAP-R	TGACTGCATTGGTACCCTCC		
*pgaA*	PGA-F	GCTGAAGCTCAAGATGTGGC	91	This study
	PGA-R	ATGCAACCCGTACCAACTGA		
*abaI*	*abaI*-F *abaI*-R	GTACAGTCGACGTATTTGTTGAATATTTGGG CGTACGTCTAGAGTAATGAGTTGTTTTGCGCC	382	[44]
